# Emerging Immunotherapies for Advanced Non-Small-Cell Lung Cancer

**DOI:** 10.3390/vaccines13020128

**Published:** 2025-01-27

**Authors:** Emily Wolf, Guilherme Sacchi de Camargo Correia, Shenduo Li, Yujie Zhao, Rami Manochakian, Yanyan Lou

**Affiliations:** Division of Hematology and Medical Oncology, Mayo Clinic, 4500 San Pablo Rd, Jacksonville, FL 32224, USA; butts.emily@mayo.edu (E.W.); correia.guilherme@mayo.edu (G.S.d.C.C.); li.shenduo@mayo.edu (S.L.); zhao.yujie@mayo.edu (Y.Z.); manochakian.rami@mayo.edu (R.M.)

**Keywords:** non-small-cell lung cancer, immune checkpoint inhibitors, immunotherapy, antibody–drug conjugate, bispecific antibodies, monoclonal antibodies, cancer vaccines, cellular therapy, tumor-infiltrating lymphocytes, gut microbiome

## Abstract

Lung cancer remains the leading cause of cancer-related mortality worldwide. Non-small-cell lung cancer (NSCLC) is the most common type of lung cancer, with nearly half of all patients diagnosed at an advanced stage. Immune checkpoint inhibitors (ICIs) harness the host immune system to combat malignant cells. ICIs, which target programmed death-ligand 1 (PD-L1), programmed cell death 1 (PD-1), and cytotoxic T-cell lymphocyte-4 (CTLA-4), have transformed the treatment landscape for advanced NSCLC. While a subset of patients experiences a long-term durable response, most patients will develop disease progression. New drugs targeting novel pathways are being tested in clinical trials to improve the efficacy of immunotherapy and overcome resistance patterns. This review aims to summarize the currently available ICIs for advanced NSCLC and describe emerging immunotherapies with recently published data from phase I/II clinical trials.

## 1. Introduction

Globally, lung cancer remained the most frequently diagnosed cancer in 2022, responsible for nearly 2.5 million cases. Lung cancer is also the leading cause of cancer-related mortality worldwide [1]. NSCLC comprises over 80% of all lung cancer cases, with adenocarcinoma and squamous-cell carcinoma representing the most common histologic subtypes [2]. Despite improved screening rates in high-risk patients, most patients are diagnosed with advanced stage disease. Overall lung cancer mortality is declining in economically developed countries, likely due both to a reduction in incidence and improved survival related to advancement in therapies [3]. There remains no cure for metastatic lung cancer.

The current management of metastatic NSCLC is personalized, driven by histology and comprehensive molecular profiling utilizing next-generation sequencing. Many lung adenocarcinomas have an actionable driver mutations involving genes such as *epidermal growth factor receptor* (*EGFR*), *anaplastic lymphoma kinase* (*ALK*), *Kirsten rat sarcoma virus* (*KRAS*), *MET*, *c-ros oncogene 1* (*ROS1*), *HER2*, *BRAF*, *RET*, and *NTRK* [4]. In addition to the advent of targeted therapies for specific driver mutations, the development of immune checkpoint inhibitor (ICI) therapy has revolutionized the treatment of NSCLC.

The discovery of cytotoxic T lymphocyte-associated molecule-4 (CTLA-4), programmed death-ligand 1 (PD-1), and programmed cell death receptor-1 ligand (PD-L1) pathways has paved the way for the development of ICI therapy, which has significantly improved the survival in patients with NSCLC without EGFR or ALK alterations. CTLA-4 is upregulated on the surface of active T cells as an inhibitory mechanism, preventing T-cell receptor (TCR) signaling by competing with the costimulatory molecule CD28 for binding to B7-1 and B7-2 ligands [5]. CTLA-4 functions to dampen T-cell activation and is, therefore, critical for immune tolerance [5]. PD-1 is upregulated upon the activation of T and B lymphocytes and serves to regulate T-cell activation through interaction with PD-L1 and PD-L2 [5]. When PD-1 binds to the PD-L1 and PD-L2 ligands, PD-1 transmits a negative costimulatory signal to attenuate T-cell activation [5]. Blocking CTLA-4, PD-L1, and PD-1 has been shown to mediate an anti-tumor response [6].

The first immunotherapy drug approved by the Food and Drug Administration (FDA) for lung cancer was nivolumab (anti-PD-1) in 2015. The CheckMate-017 phase III clinical trial randomized patients with advanced squamous NSCLC who had disease progression during or after first-line chemotherapy to receive either nivolumab or docetaxel [7]. Nivolumab led to superior overall survival (OS), response rate, and progression-free survival (PFS). The median OS (mOS) was 9.2 months with nivolumab versus 6.0 months with docetaxel. The benefit was seen regardless of PD-L1 status [7]. Pembrolizumab (anti-PD-1) was the first immunotherapy to gain FDA approval in the first-line setting. The KEYNOTE-024 phase III clinical trial randomized patients with untreated advanced NSCLC to either pembrolizumab or chemotherapy. In patients with a high PD-L1 expression (tumor proportion score ≥ 50%), single-agent pembrolizumab was associated with significant PFS and OS benefits compared with chemotherapy [8]. More recent studies demonstrated that pembrolizumab in combination with chemotherapy led to significant survival benefits regardless of PD-L1 expression in both squamous and non-squamous histologic subtypes [9,10]. Since then, multiple other ICIs have been FDA-approved for metastatic NSCLC and are summarized in Table 1. A large study reporting real-world outcomes in patients who received first-line immunotherapy plus chemotherapy showed an mOS of 10.6 months in patients with squamous NSCLC and 12.0 months in patients with non-squamous subtypes [11].

Despite the encouraging long-term durability of responses seen in a subset of patients receiving ICI therapy, there remains a proportion of patients who do not respond to ICI due to either primary or secondary resistance. Primary resistance is progressive disease after 6 weeks but less than 6 months of ICI treatment [28]. Primary resistance occurs due to intrinsic adaptive changes in the cancer cells or the extrinsic modulation of the tumor microenvironment, representing an innate inability of the immune system to activate an appropriate immune response. Secondary resistance, on the other hand, occurs after the initial benefit from ICI and is defined as disease progression within 12 weeks from the last ICI dose [28]. Tumor cells can acquire specific genetic alterations which allow them to evade the immune response.

There is an essential need for newer drugs to overcome and prevent the resistance patterns seen with ICI therapy as well as targeting novel pathways to improve the efficacy of immunotherapy. This paper reviews the most recently published data on emerging immunotherapies for NSCLC. A literature search was conducted utilizing American Society of Clinical Oncology (ASCO) meeting abstracts, American Association for Cancer Research (AACR) meeting abstracts, PubMed, and ClinicalTrials.gov to identify phase I/II, first-in-human clinical trials for advanced or metastatic NSCLC from December 2023 to June 2024. We focused on immunotherapies targeting novel pathways, including antibody–drug conjugates, bispecific antibodies, monoclonal antibodies, microbiome-based therapeutics, cellular therapies, and cancer vaccines. Each study was individually reviewed and is summarized in Table 2.

## 2. Antibody–Drug Conjugate

### Brentuximab Vedotin

**Figure 1:**
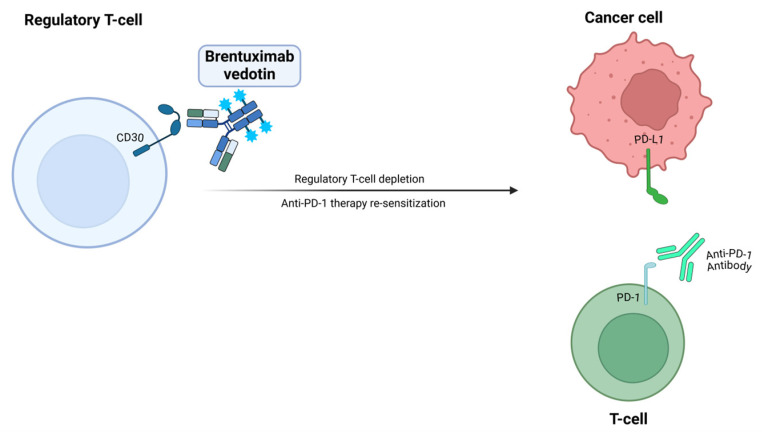
Illustration representing the mechanism of action of BV. BV depletes CD30^+^ regulatory T cells, enhancing the activity of cytoxic T cells, and promoting the re-sensitization of anti-PD-1 therapy.

Brentuximab vedotin (BV) is an antibody–drug conjugate medication that is currently approved for several types of CD30^+^ lymphoma including classical Hodgkin lymphoma and anaplastic large-cell lymphoma. It is composed of an anti-CD30 monoclonal antibody (mAb) conjugated to monomethyl auristatin E (MMAE), an anti-microtubule agent [54]. It is not currently approved for any solid-tumor malignancy. Pre-clinical studies have reported that BV can selectively deplete CD30-expressing regulatory T cells, enhancing cytotoxic T lymphocyte activity (Figure 1) [55]. Thus, BV is an attractive therapy to combine with ICIs. BV, in combination with pembrolizumab, is currently undergoing investigation via a phase II trial in patients with previously treated metastatic NSCLC or cutaneous melanoma [31]. In this study, 55 treatment refractory patients with NSCLC were enrolled, and the median prior lines of therapies was three. At a median follow-up of 15.4 months, the median PFS (mPFS) was 4.1 months, and the mOS was 13.9 months in the NSCLC cohort [31]. Improved cytotoxic T-cell infiltration was noted in 11 of 19 patients, supporting the immunomodulatory effects of BV when combined with pembrolizumab [31]. Peripheral neuropathy was a major side effect, reported in 48% of patients [31].

## 3. Bispecific Antibody

### 3.1. CD-16 and EGFR

**Figure 2:**
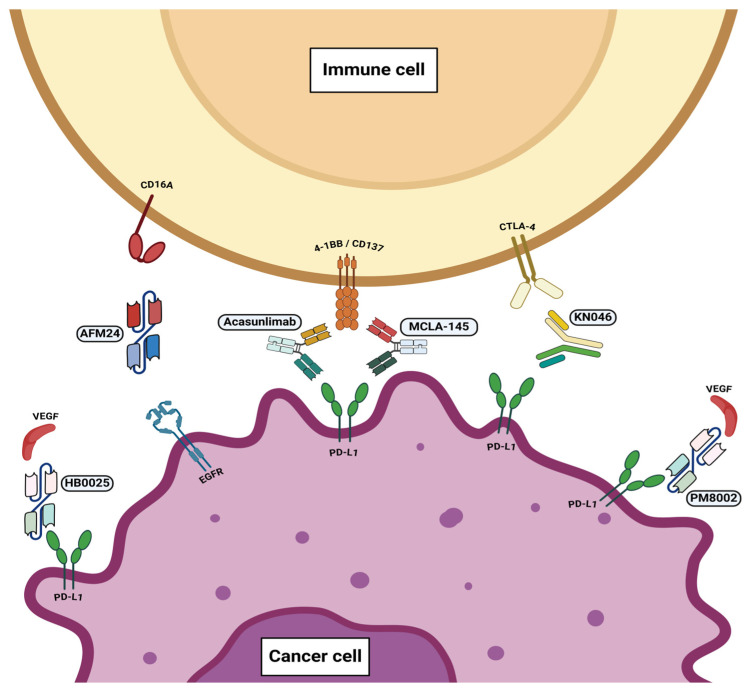
Illustration demonstrating the mechanism of action of bispecific antibodies, HB0025, AFM24, acasunlimab, MCLA-145, KN046, and PM8002.

AFM 24 is a first-in-class, bispecific, IgG1-scFv fusion antibody targeting CD16A on innate immune cells and EGFR on tumor cells (Figure 2) [56]. In preclinical studies, AFM24 demonstrated antibody-dependent cell-mediated cytotoxicity via natural killer (NK) cells and cellular phagocytosis via macrophages [56]. An ongoing phase I/IIa study is investigating the combination of AFM24 with atezolizumab, a PD-L1 inhibitor, in patients with advanced or metastatic EGFR wild-type (EGFR-WT) NSCLC who have progressed on chemotherapy and ICI [30]. As of January 2024, there were 15 response-evaluable patients. One patient had a confirmed complete response (CR) and three patients had confirmed partial response (PR) [30]. Seven patients achieved stable disease (SD) [30]. The most common adverse event (AE) was infusion-related reaction with two grade 3 reactions reported [30]. The clinical trial is currently ongoing with plans to enroll up to 40 patients in this cohort.

### 3.2. PD-L1 and 4-1BB

4-1BB is a costimulatory receptor belonging to the TNF receptor family [57]. 4-1BB agonists have been shown to both promote an anti-tumor and anti-viral response while also alleviating autoimmune disease, thus making it an attractive target for immunooncology [57]. Acasunlimab (DuoBody^®^-PD-L1x4-1BB) is a first-in-class, bispecific antibody therapy that blocks PD-L1 and activates 4-1BB in a manner dependent on simultaneous PD-L1 binding (Figure 2) [58]. A phase II study is investigating acasunlimab as a monotherapy and in combination with pembrolizumab in patients with metastatic NSCLC [34]. Patients with PD-L1^+^ metastatic NSCLC with progression after at least one prior ICI were randomized to receive acasunlimab monotherapy (arm A), acasunlimab with pembrolizumab 200 mg every 3 weeks (arm B), or acasunlimab with pembrolizumab every 6 weeks (arm C) [34]. Among 63 patients, the confirmed objective response rate (ORR) was 13%, 21%, and 22%, while the six-month PFS rate was 0%, 18%, and 33%, for arms A, B, and C, respectively [34]. Previously, 4-1BB agonist clinical development has been limited by liver toxicity. Liver-related events with grade ≥ 3 were 13.3% in the combination arms [34].

### 3.3. PD-L1 and CTLA-4

KN046 is a recombinant, humanized bispecific antibody that blocks the binding of PD-L1 to PD-1 and CTLA-4 to CD80/CD86 (Figure 2). In a phase II, multicenter trial, patients with metastatic NSCLC received KN046 in combination with chemotherapy in the first-line setting [35]. With a median follow-up time of 23.1 months in 87 enrolled patients, the ORR was 46%, and the median duration of response was 8.1 months [35]. The mPFS was 5.8 months, and the mOS was 26.6 months. Grade ≥ 3 treatment-related adverse events (TRAEs) occurred in 66.7% of patients [35]. The most common TRAEs were anemia, loss of appetite, neutropenia, leukopenia, and thrombocytopenia [35]. A total of 17 patients discontinued treatment due to treatment-emergent adverse events (TEAEs), and treatment-related deaths occurred in 4 patients [35]. A phase III, randomized, controlled trial is ongoing to verify the findings.

### 3.4. PD-L1 and VEGF

Vascular endothelial growth factor (VEGF) is critical for angiogenesis by promoting the growth of vascular endothelial cells [59]. Blocking the binding of VEGF to VEGF receptor (VEGFR) is thus expected to inhibit angiogenesis and tumor growth [60]. HB0025 is a bispecific antibody targeting PD-L1 and VEG-F, formed via the fusion of domain 2 of VEGFR1 and anti-PD-L1 mAb (Figure 2) [60]. A phase I study evaluated the safety and efficacy of HB0025 in patients with advanced solid tumors [36]. A total of 12 patients with advanced, heavily pretreated NSCLC (with a median of four prior treatment lines) received HB0025 at various doses: 3 mg/kg (*n* = 1), 6 mg/kg (*n* = 2), 10 mg/kg (*n* = 2), 12 mg/kg (*n* = 1), 20 mg/kg (*n* = 5), and 30 mg/kg (*n* = 1). The ORR was 25% and the disease control rate (DCR) was 66.7%, including three PR, five SD, and four progressive disease (PD) [36]. Grade ≥ 3 TRAEs occurred in two patients (16.77%), and the most common TRAEs reported were proteinuria, lymphocytopenia, hyperbilirubinemia, and hypertension. Importantly, there was no TRAE which led to drug discontinuation [36]. Phase II studies of HB0025 in combination with chemotherapy are ongoing [36].

PM8002 is a bispecific antibody targeting PD-L1 and VEGF-A (Figure 2). A phase Ib/IIa trial investigated the safety and efficacy of PM8002 in patients with advanced NSCLC. The study included previously untreated patients with EGFR/ALK wild type and PD-L1+, patients with EGFR mutations who had failed prior EGFR-TKI treatment, and wild-type EGFR/ALK who had failed anti-PD-1/L1 therapy and platinum-based chemotherapy regimens [37]. Among all cohorts, 61 patients were evaluated with 16 (26.2%) PR and 32 (52.4%) SD observed [37]. Grade ≥ 3 TRAEs occurred in 18% of patients, and 8.2% of patients discontinued PM8002 due to TRAEs [37].

### 3.5. CD137 and PD-L1

MCLA-145 is a bispecific antibody targeting CD137 and PD-L1. MCLA-145 binds to PD-L1 on a tumor cell or antigen-presenting cell and CD-137 expressed on a T-effector cell (Figure 2) [61]. The upregulation of CD137 leads to the activation of the NF-kB signaling pathway, enhancing T-cell differentiation, expansion, and activation [61]. A phase I study investigated MCLA-145 as monotherapy or in combination with pembrolizumab in patients with advanced or metastatic tumors with PD-L1 > 1% [38]. Of the 72 patients treated, 25% had NSCLC. The recommended dose for expansion was determined to be 40 mg every three weeks for both monotherapy and combination therapy [38]. Overall, the DCR was 37% with monotherapy and 68% with combination therapy.

## 4. Cellular Therapy

### 4.1. T-Cell Receptor–Engineered T Cells

KRAS is the most frequent gene mutation identified in human cancer, with more than 30% of lung adenocarcinomas bearing KRAS-activating mutations [62]. Currently, there are two FDA-approved targeted therapies for KRAS G12C mutation, sotorasib and adagrasib, indicated in the second-line setting. There are no approved therapies for KRAS G12V. FH-A11KRASG12V-TCR is an autologous CD8+ and CD4+ transgenic T-cell product expressing KRASG12V mutation-specific T-cell receptors [39]. A first-in-human phase I trial aims to study the safety, tolerability, maximum tolerated dose, and preliminary anti-tumor activity of FH-A11KRASG12V-TCR. The study is ongoing and currently recruiting patients with advanced pancreatic cancer, colorectal cancer, and NSCLC [39].

### 4.2. Tumor-Infiltrating Lymphocytes

**Figure 3:**
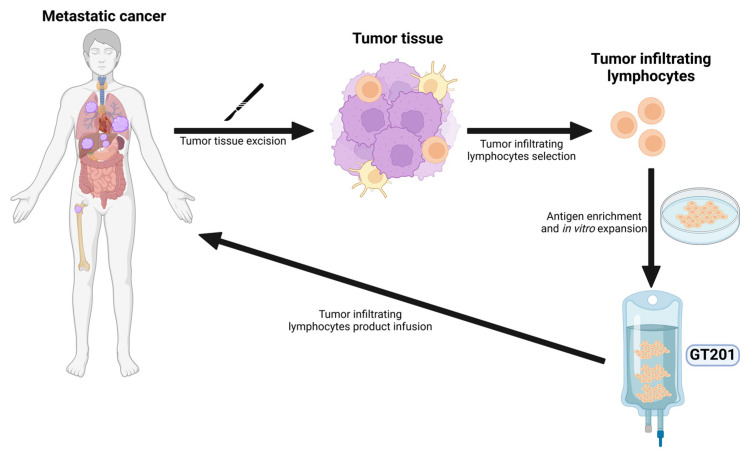
Illustration demonstrating the mechanism of action of tumor-infiltrating lymphocytes. Lymphocytes isolated from the patient’s tumor are expanded in vitro. After treatment with non-myeloablative lymphodepletion chemohterapy, patients receive an infusion of TILs followed by IL-2, which enhances the expansion of TILs and the subsequent anti-tumor response.

Tumor-infiltrating lymphocytes (TILs) are a type of adoptive cellular therapy. Natural infiltrating lymphocytes are isolated from tumor tissues and expanded in vitro. Patients receive non-myeloablative lymphodepletion chemotherapy followed by the infusion of TILs and a high dose of IL-2, augmenting the expansion of cells and antitumor response [63]. Lifileucel, a TIL therapy, was granted accelerated approval by the FDA for unresectable or metastatic melanoma in February 2024. GT201 is an engineered TIL expressing a membrane-bound IL-15 with preclinical studies showing enhanced antitumor reactivity (Figure 3) [64]. A phase I, open-label, single-arm study investigated the safety and efficacy of G201 in patients with advanced solid tumors [40]. Results are reported for a total of seven patients including three patients with NSCLC. Among the seven-patient cohorts, three patients (42.9%) had a PR, and two patients (28.6%) had SD. Disease control, defined as SD ≥ 24 weeks or PR, was achieved in all three patients with NSCLC [40]. The study is actively recruiting with an estimated enrollment of 26 patients.

## 5. Gut Microbiome

Research has shown that the gut microbiome plays an important role in the innate and adaptive immune response and influences the efficacy of cancer treatments, including immunotherapy and chemotherapy [65]. The role of the gut microbiome of patients with advanced NSCLC was studied by analyzing 71 stool samples from patients prior to treatment with immune checkpoint blockade [66]. Ren et al. found a significant association between elevated gut microbiota diversity and response to treatment with ICIs [66]. There are many ongoing trials investigating fecal microbiota transplant (FMT) and live bacterial consortium in patients with solid tumors. BMC128 is a live bacterial consortium composed of four commensal bacterial strains [41]. A first-in-human open-label phase I study investigated the safety and tolerability of BMC128 in combination with nivolumab [41]. Twelve patients, including five with NSCLC (wild-type EGFR/ALK) who previously progressed on PD1/PDL-1 inhibitors, were recruited [41]. Prior to treatment initiation, patients were given antibiotics for the depletion of native microbiota. Induction therapy consisted of BMC128 monotherapy daily for 14 days, which was followed by daily BMC128 with nivolumab administered every 28 days. No serious adverse events (SAEs) were reported. Preliminary efficacy data demonstrated clinical benefit for the first four patients with stable disease beyond the first imaging time point. A phase II study to further investigate efficacy is planned [41]. Additionally, a phase I/II trial of healthy donor FMT is estimated to start recruiting at the end of 2024 [42]. This trial will study responder-derived FMT in combination with pembrolizumab in patients with relapsed or refractory PD-L1-positive NSCLC. Donors are advanced cancer patients who have maintained a durable remission with PD-1 monotherapy.

## 6. Monoclonal Antibodies

### 6.1. Anti-ILT4 and Anti-ILT3

**Figure 4:**
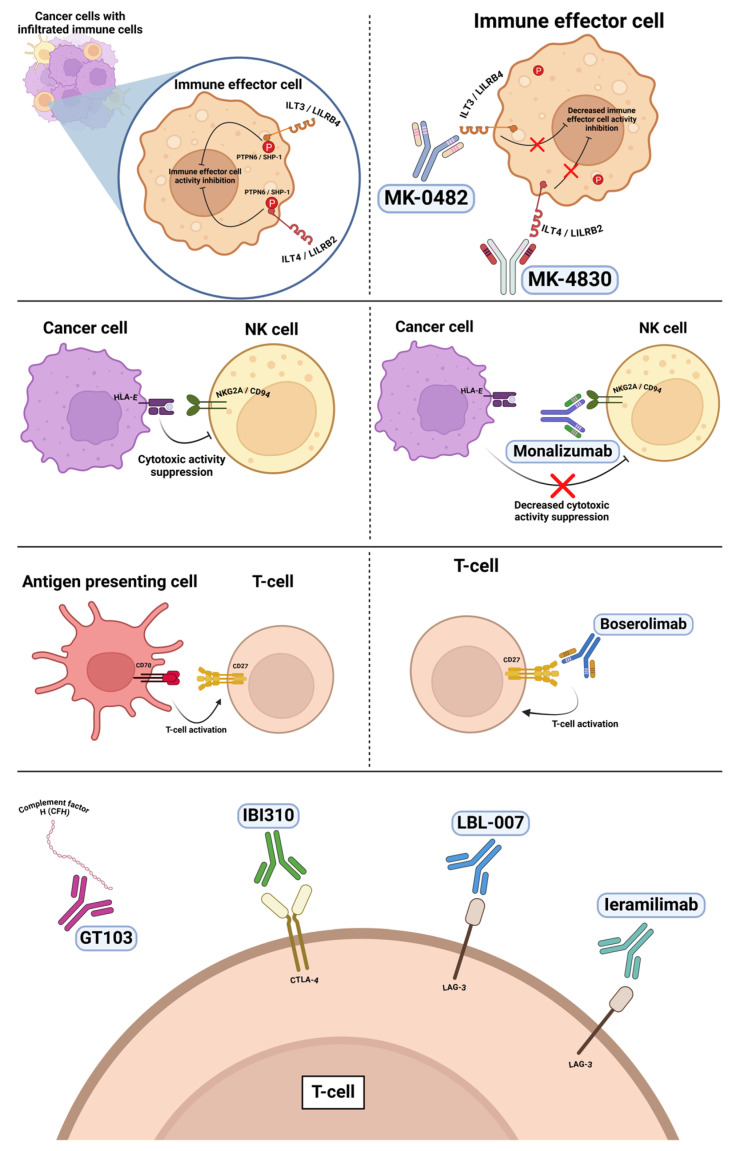
Summary of monoclonal antibodies and mechanism of action. MK-4830 is an anti-ILT4 mAb, while MK-0482 is an anti-ILT3 mAb. MK-4830 and MK-0482 block immunosuppressive activity of ILT-4 and ILT-3 receptors. Monalizumab is an anti-NKG2A mAb, which attenuates an immunsuppressive receptor on NK cells. Boserolimab is an anti-CD27 mAb, inhibiting the ability of CD27 to function as a costimulatory molecule. LBL-007 and leramilimab are mAbs targeting LAG-3, an inhibitory receptor found on T cells. IBI310 is an anti-CTLA-4 mAb. GT103 is an anti-CFH mAb, which increased activation of the alternative complement pathway.

Immunoglobulin-like transcript 4 (ILT4), which functions as an immunosuppressive receptor, is expressed in dendritic cells, monocytes, macrophages, and neutrophils [67]. Increased ILT4 expression in tumor cells of lung adenocarcinoma was associated with reduced T-cell infiltration of the tumor microenvironment and worse patient prognosis [67]. MK-4830 is a first-in-class anti-ILT4 mAb which was shown to inhibit tumor growth in a humanized mouse model (Figure 4) [68]. The KEYMAKER-U01 substudy is a phase II umbrella study investigating the combination of pembrolizumab with investigational agents [43]. Arm B evaluated pembrolizumab in combination with MK-4830 and included 45 patients with NSCLC who progressed on or after prior ICI therapy and platinum-based chemotherapy [43]. ORR was 11% with 1 CR and 4 PR. mPFS was 2.4 months. Grade ≥ 3 AEs occurred in 25 patients (56%) with eight (18%) discontinuing treatment due to any AE [43].

Immunoglobulin-like transcript 3 (ILT3), like ILT4, is an immunosuppressive receptor, expressed in dendritic cells and monocytes. ILT3 knockdown in dendritic cells stimulates stronger T-cell responses to viral antigens and alloantigens with enhanced cytokine and chemokine production [69]. MK-0482 is an anti-ILT3 antibody that was studied in combination with pembrolizumab in the KEYMARKER-U01 study (arm C) (Figure 4) [43]. A total of 45 patients with NSCLC who progressed on or after prior ICI therapy and platinum-based chemotherapy received MK-0482 and pembrolizumab. ORR in this arm was 4% with mPFS of 2.6 months. Grade ≥ 3 AEs occurred in 17 patients (38%) with two patients discontinuing treatment due to any AE [43].

### 6.2. Anti-CD27

CD27 is a member of the TNF receptor family and functions as a B- and T-cell costimulatory molecule dependent upon CD70 expression. CD70 is transiently expressed on antigen-activated B and T cells, NK cells, and dendritic cells [70,71]. Boserolimab is an anti-CD27 monoclonal antibody which was studied in combination with pembrolizumab in the KEYMARKER-U01 study (arm A) (Figure 4). Arm A included 37 patients with NSCLC who progressed on or after prior ICI therapy and platinum-based chemotherapy. ORR was 8% with one CR and two PRs. mPFS was 2.4 months. Grade ≥ 3 AEs occurred in 21 patients (57%) with six patients discontinuing treatment due to any AE [43].

### 6.3. Anti-NKG2A

NKG2A, also known as CD94, is a receptor expressed on NK cells and activated CD8^+^ T cells [72]. Blocking antibodies to NKG2A results in the inhibition of an immune checkpoint. Monalizumab is a humanized anti-NKG2A blocking mAb (Figure 4) [72]. A phase I/II study investigated monalizumab in combination with durvalumab in patients with advanced solid tumors [44]. The study enrolled a total of 185 patients, including 20 patients with NSCLC who had received at least one prior line of therapy. In the NSCLC cohort, there were two (10%) PRs and six (30%) SDs observed. mPFS was 1.9 months with mOS of 8.8 months. No dose-limiting toxicities were reported [44].

### 6.4. Anti-Complement Factor H

The alternative complement pathway is a part of the innate immune system triggered by C3b binding to microbes or IgG antibodies. Complement factor H (CFH) is an important regulatory protein that defends cells from complement activation [45]. GT103 is a first-in-class fully human-derived IgG3 anti-CFH monoclonal antibody that increases activation of alternative complement pathway with increased deposition of C3b and was shown to enhance anti-tumor activity in preclinical models (Figure 4) [45]. A phase Ib, first-in-human study evaluated GT103 in patients with NSCLC who have previously received ICIs and a platinum-based chemotherapy. A total of 31 patients were enrolled. Stable disease was observed in nine patients (29%). mPFS was 6 weeks with a 24-week PFS of 9.7%. mOS was 25.7 weeks with a 24-week OS of 50.6%. Grade ≥ 3 TRAEs occurred in three patients, including acute kidney injury, decreased lymphocyte count, and anemia. A phase II trial evaluating the combination of GT103 and pembrolizumab is currently enrolling patients [45].

### 6.5. Anti-CTLA-4

IBI310 is a recombinant fully human IgG1 anti-CTLA-4 antibody (Figure 4) [46]. Sintilimab is a recombinant fully human IgG4 anti-PD-1 monoclonal antibody. Combining CTLA-4 blockade with anti-PD1 therapy is a strategy aiming to overcome adaptive resistance to anti-PD-1 treatment. A phase Ib clinical trial evaluated the safety and efficacy of IBI310 with sintilimab in patients with NSCLC who progressed following anti-PD-(L)-1. Patients were randomized to receive a lower dose of IBI310 (1 mg/kg every 3 weeks (Q3W), cohort A) or a higher dose of IBI310 (3 mg/kg Q3W, cohort B) both in combination with sintilimab. The ORR was 0% and 13.3% in cohort A and B, respectively. The DCR was 46.7% and 66.7% in cohort A and B, respectively. Two patients in cohort B had a PR to treatment. Grade ≥ 3 TRAEs occurred in 40% and 53.3% of patients in cohorts A and B, respectively. The most common TRAEs were anemia, elevated liver enzymes, rash, hypoalbuminemia, and thrombocytopenia. Importantly, there were three treatment-related deaths due to immune-related hepatitis, immune-related myocarditis, and another patient who died of an unknown cause [46].

### 6.6. Anti-LAG-3

Lymphocyte activation gene 3 (LAG-3), or CD223, is an inhibitory receptor that plays an important role in the prevention of autoimmunity. The biological function of LAG-3 is to balance co-stimulatory receptor activity and limit T-cell activation [73]. LAG-3 is thus an attractive immunotherapy target. LBL-007 is a novel fully human IgG4 anti-LAG-3 antibody (Figure 4). A phase Ib/II clinical trial investigated LBL-007 in combination with toripalimab (anti-PD-1 antibody) in patients with advanced solid tumors [47]. In the phase Ib dose escalation study, patients received either LBL-007 at 200 or 400 mg, both in combination with toripalimab at 240 mg (both received Q3W). For the dose expansion part of the study, patients received LBL-007 at 400 mg and toripalimab Q3W. Out of a total of 75 evaluable patients, ORR and DCR were 13.3% (10/75) and 48.0% (36/75), respectively. The response rate in NSCLC patients is not reported. Grade ≥ 3 TEAEs occurred in 19 patients (23.8%) with six patients (7.5%) discontinuing treatment due to TEAEs [47].

Ieramilimab (LAG525) is a humanized anti-LAG-3 monoclonal antibody (Figure 4). A phase II multicenter open-label clinical trial investigated ieramilimab in combination with spartalizumab (anti-PD-1 antibody) [48]. Patients with advanced solid tumors were grouped depending on prior ICI therapy and received ieramilmab 400 mg and spartilizumab 300 mg Q3W. A total of 42 patients with NSCLC were enrolled. In patients with NSCLC naïve to ICI therapy, the ORR was 15% with three (15%) being PR and seven (35%) being SD observed. In patients with NSCLC pretreated with ICI therapy, the ORR was 0% with 11 (50%) SD observed. The most frequent AE suspected to be related to the study drug included fatigue, rash, and nausea. SAEs suspected to be treatment-related occurred in 8.5% and 2.2% of patients in the ICI naїve and pretreated cohorts, respectively [48].

## 7. Other Molecules

### 7.1. CXCR2 Antagonist

C-X-C motif chemokine receptor 2 (CXCR2) expression is upregulated in several cancer types including NSCLC. In vitro, CXCR2 was found to activate the JAK2/STAT3 signaling pathway and promote cell proliferation and invasion while suppressing cell apoptosis [74]. CXCR2 tumor expression correlated with advanced stage, poor pathologic differentiation, and worse OS in NSCLC patients [74]. Navarixin is a CXCR2 antagonist. A phase II study randomized patients with previously treated prostate cancer, colorectal cancer, or NSCLC to receive either navarixin 30 or 100 mg orally once daily plus pembrolizumab 200 mg intravenously every 3 weeks up to 35 cycles [49]. A total of 25 patients with NSCLC were enrolled. The ORR in patients with NSCLC was 0%. Dose-limiting toxicities occurred in 2/48 (4%) and 3/48 (6%) of patients receiving navarixin 30 mg and 100 mg, respectively [49]. Despite promising preclinical results with navarixin, this trial did not demonstrate adequate efficacy.

### 7.2. TLR 7/8 Agonist

Toll-like receptors (TLRs) are a family of pattern recognition receptors, which trigger downstream signaling, activating both the innate and adaptive immune system. TLR 7 and 8 are expressed in the intracellular endosomes and recognize nucleic acids, generating an immune response [75]. EIK1001 is a TLR7/8 agonist, which activates myeloid and plasmacytoid dendritic cells [50]. A phase I study investigated EIK1001 as either monotherapy or in combination with pembrolizumab in advanced solid tumors [76]. The patients were heavily pretreated with a median of three prior treatment regimens. A total of 50 patients were efficacy-evaluable with 3/50 (6%) being CR, 4/50 (8%) being PR, and 24/50 (48%) being SD. A total of 9/51 patients (17.6%) experienced grade ≥ 3 TRAE, with one patient discontinuing therapy due to cytokine release syndrome. There were no treatment related deaths observed [76]. Overall, this study showed promising efficacy of EIK1001 in combination with pembrolizumab. A phase II multicenter open-label study investigating EIK1001 in combination with pembrolizumab and chemotherapy in patients with metastatic NSCLC opened in January 2024 [50]. Unlike the prior phase I study, this study will evaluate EIK1001 in the first-line setting. The primary objectives of the study are to determine safety and tolerability, with secondary objectives including ORR and duration of response (DOR) [50].

### 7.3. α/β IL-2R Agonist

Interleukin-2 (IL-2) is a potent cytokine with the dual function of stimulating effector T-cell expansion and differentiation while also inducing tolerance by promoting differentiating of regulatory T-cells [77]. IL-2 induces its immune response by binding to IL-2R [77]. High-dose recombinant IL-2 became the first FDA-approved immunotherapy when it was approved for the treatment of metastatic renal cell carcinoma in 1992 [77]. The clinical utility of IL-2 therapy for cancer has been limited by its short half-life of less than 30 min as well as major toxicities including vascular leak syndrome, pulmonary edema, and hypotension [78]. STK-012 is a first-in-class CD25/CD122-selective IL-2 mutein, engineered to stimulate effector T-cells and avoid systemic NK and naïve T-cell activation [79]. A phase I study investigated STK-012 in patients with advanced relapsed or refractory solid tumors with NSCLC as the most common tumor type in 35.6% of patients [51]. Of 45 patients treated at 7 dose levels across 2 schedules (every week (QW) at 0.375 mg and 0.75 mg; Q3W at 0.75−3mg), no dose-limiting toxicities were observed [51]. Grade ≥ 3 TRAEs occurred in 12/45 of patients (26.6%). Notably, no subjects developed capillary leak syndrome, and less than 5% of patients developed other IL-2-associated TRAEs, including hypotension and peripheral edema. A total of 38 patients were evaluated for efficacy with 3 PR and 17 SD observed. Phase Ib dose expansions in relapsed refractory NSCLC are ongoing [51].

## 8. Vaccines

### 8.1. CCL21-DC

**Figure 5:**
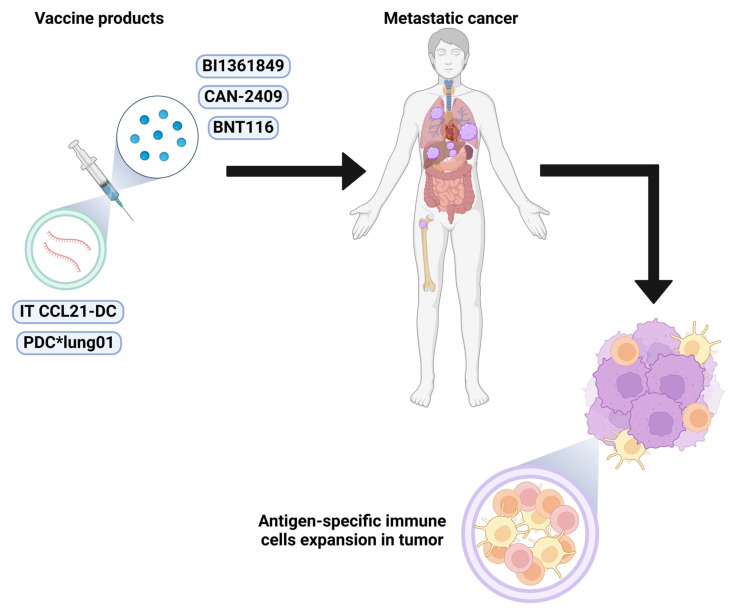
Illustration depicting the mechanism of investigational cancer vaccines, BI1361849, CAN-2409, BNT116, CCL21-DC, and PDC*lung01. BI1361849, CAN-2409, and BNT116 contain tumor associated antigens, resulting in antigen-specific immune cell expansion in the tumor following vaccine administration. CCL21-DC is a cancer vaccine containing autologous dendritic cells transduced with an adenoviral vector expressing the *CCL21* gene (CCL21-DC). CCL21-DC is delivered via intratumoral administration. PDC*lung01 is a cancer vaccine containing an irradiated plasmacytoid dendritic cell line with HLA-A*02-01-restricted peptides including tumor antigens associated with NSCLC.

CCL21 is a chemokine involved in promoting leukocyte chemotaxis and activation. Previously, intratumoral administration of murine dendritic cells genetically modified to express CCL21 led to significant tumor response in a murine lung cancer model [80]. This work provided the rationale for a phase I clinical study evaluating intratumoral administration of autologous dendritic cells transduced with an adenoviral vector expressing the *CCL21* gene (CCL21-DC) (Figure 5) [81]. Sixteen patients with advanced NSCLC received two vaccinations of varying doses (1 × 10^6^, 5 × 10^6^, 1 × 10^7^, or 3 × 10^7^ dendtric cells/injection) via computed tomography (CT) or bronchoscopic-guided intratumoral injections. Increased CD8^+^ T-cell infiltration was found in 54% of patients, and this subset of patients was also found to have increased PD-L1 expression [81]. It was hypothesized that the tumor-mediated impairment of T-cell function could lead to a decreased anti-tumor response from CCL21-DC. A phase I study evaluating the combination of intratumoral CCL21-DC and pembrolizumab is currently recruiting patients [52]. The study plans to enroll 24 patients with dose escalation followed by dose expansion with aim of determining the maximum tolerated dose (MTD) and subsequently the objective response rate at the MTD [52].

### 8.2. PDC*lung01

PDC*lung01 is a therapeutic cancer vaccine based on an irradiated plasmacytoid dendritic cell line loaded with HLA-A*02:01-restricted peptides including NY-ESO-1, melanoma antigen gene (MAGE)-A3, MAGE-A4, multi-MAGE-A, mucin 1 (MUC1), survivin and Melan-A, which are tumor antigens associated with NSCLC (Figure 5) [53]. A phase I/II study evaluated PDC*lung01 in four cohorts of HLA-A*02-positive patients with NSCLC. A low dose and high dose of PDC*lung01 was administered to either patients with stage II/IIIA NSCLC in the adjuvant setting or in patients with stage IV NSCLC, PD-L1 ≥ 50% with no targetable mutation in the first-line setting. PDC*lung01 was administered subcutaneously and intravenously weekly for six consecutive doses, in combination with pembrolizumab 200 mg every 3 weeks. The ORR and PFS were assessed in a total of 21 patients with stage IV NSCLC who received the higher dose of PDC*lung01. The ORR was 63.2% including 12 patients (63.2%) with PR and seven patients (36.8%) with SD. The mPFS was 10.9 months. One grade 4 allergic infusion-related reaction was observed as the only severe TRAE [53].

### 8.3. CAN-2409

CAN-2409 is a replication-defective adenovirus encoding herpes simplex virus thymidine kinase (HSV-tk) gene (Figure 5) [54]. HSV-tk converts valacyclovir within the tumor microenvironment into a toxic metabolite while the adenoviral proteins provide a pro-inflammatory signal, leading to in situ vaccination against the patient’s own tumor [82]. A phase II clinical trial evaluated CAN-2409 and valacyclovir in combination with ICI therapy in patients with non-resectable advanced NSCLC who were refractory or resistant to ICI therapy. Patients received two doses of CAN-2409 via injection into the lung tumor, lymph node metastasis, or distant metastasis followed by oral valacyclovir. The mOS of the evaluable population (*n* = 44) was 22 months. A total of 65% of patients were found to have tumor shrinkage of both injected and uninjected lesions. There were no dose-limiting toxicities observed [54].

### 8.4. BNT116

BNT116 is an intravenously administered RNA-based lipoplex vaccine comprising six mRNAs including MAGE A3, CLDN6, KK-LC-1, PRAME, MAGE A4, and MAGE C1, encoding tumor antigens frequently expressed in NSCLC (Figure 5) [55]. LuCa-MERIT-1 is a first-in-human phase II trial evaluating BNT116 alone or in combination with docetaxel in patients with advanced NSCLC. Patients must have progressed on ICI and platinum-based chemotherapy. A total of 20 patients received BNT116 and docetaxel. Grade ≥ 3 TEAEs with incidence ≥ 10% included neutropenia, pneumonia, hypertension, decreased lymphocyte count, diarrhea, and fatigue. The ORR was 35% with seven patients (35%) with PR and 10 patients (50%) with SD [55].

### 8.5. BI1361849

BI1361849 is an RNA-based cancer vaccine composed of six mRNAs including MUC1, survivin, NY-ESO-1, 5T4, MAGE-C2, and MAGE-C1 [56]. A phase Ib study evaluated BI1361849 and durvalumab with or without tremelimumab for patients with NSCLC. Patients were randomized to arm A (BI1361849 + durvalumab) or arm B (BI1361849 + durvalumab + tremelimumab). The recommended combination dosages were durvalumab at 1500 mg, BI1361849 at 960 μg, and tremelimumab at 75 mg. The treatment discontinuation rate was reported as 22−24% for either arm. The ORR was 29% in arm A and 11% in arm B. PFS was 5.7 months for arm A and 2.5 months for arm B. OS was not reached for arm A and 10 months for arm B. In this study, both regimens were overall well tolerated; however, tremelimumab was not found to add clinical benefit [56].

## 9. Conclusions

The field of lung cancer immunotherapy is advancing rapidly, with innovative approaches showing promise in overcoming the challenges of immune evasion and treatment resistance. While ICIs have revolutionized the clinical practice, ongoing research is investigating diverse approaches, including antibody–drug conjugates, bispecific antibodies, monoclonal antibodies, microbiome-based therapeutics, cellular therapies, cancer vaccines, and others. These emerging therapies offer the potential to minimize off-target toxicity, improve response rates, and prolong survival for patients with lung cancer. Nonetheless, significant challenges remain, such as optimizing scientifically grounded combination therapies, identifying accurate predictive biomarkers, adopting personalized approaches, and managing immune-related adverse events. Continued research is essential to unlock the full potential of immunotherapy and ultimately improve patient outcomes in this devastating disease.

## Figures and Tables

**Table 1:** Current FDA-approved immunotherapy for advanced NSCLC.

Drug	Class	Trials	Indications
Nivolumab	Anti-PD-1	CHECKMATE-057 [12] CHECKMATE-017 [13]	Metastatic, progression on or after platinum-based chemotherapy *
Nivolumab + Ipilimumab	Anti-PD-1 Anti-CTLA-4	CHECKMATE-227 [14] CHECKMATE-9LA [15]	Metastatic, PD-L1 expression ≥ 1% with no EGFR or ALK tumor aberrations, first-line treatment Metastatic, given with 2 cycles of platinum-doublet chemotherapy, no EGFR or ALK tumor aberrations, first-line treatment
Pembrolizumab	Anti-PD-1	KEYNOTE-010 [16] KEYNOTE-042 [17] KEYNOTE-407 [18] KEYNOTE-189 [19]	Metastatic, PD-L1 expression ≥ 1%, with disease progression on or after platinum-based chemotherapy * Metastatic, PD-L1 expression ≥ 1%, first-line treatment with no EGFR or ALK tumor aberrations Stage 3, patients not candidates for surgical resection or definitive chemoradiation Metastatic, squamous, without EGFR or ALK aberrations, first-line treatment in combination with carboplatin and either paclitaxel or paclitaxel protein-bound Metastatic, non-squamous without EGFR or ALK aberrations, first-line treatment in combination with pemetrexed and platinum chemotherapy
Atezolizumab	Anti-PD-L1	IMpower110 [20] IMpower150 [21] IMpower130 [22] OAK [23]	Metastatic, PD-L1 expression > 50%, first-line treatment without EGFR or ALK aberrations Metastatic, non-squamous, no EGFR or ALK aberration, first-line in combination with bevacizumab, paclitaxel, and carboplatin Metastatic, non-squamous, no EGFR or ALK aberrations, first-line in combination with paclitaxel protein-bound and carboplatin Metastatic, disease progression during or following platinum-containing chemotherapy *
Durvalumab	Anti-PD-L1	PACIFIC [24]	Stage 3, unresectable, post-chemoradiation consolidation therapy
Durvalumab + Tremelimumab	Anti-PD-L1 Anti-CTLA-4	POSEIDON [25]	Metastatic, PD-L1 expression < 1%, no EGFR or ALK tumor aberrations, in combination with platinum-based chemotherapy
Cemiplimab	Anti-PD-1	EMPOWER-LUNG 3 [26] EMPOWER-LUNG1 [27]	Metastatic or locally advanced and not candidate for surgical resection or chemoradiation, no EGFR, ALK or ROS1 aberration, first-line, in combination with platinum-based chemotherapy Metastatic or locally advanced and not candidate for surgical resection or chemoradiation, no EGFR, ALK, or ROS1 aberration, PD-L1 expression ≥ 50%, first-line as single agent

* Patients with EGFR or ALK tumor aberrations should have disease progression on FDA-approved therapy prior to receiving therapy.

**Table 2:** Summary of early phase clinical trials.

Target	Drug	Trial	Phase	Type of Tumor	Preliminary Efficacy	Safety	Comments	**Ref**
**Antibody–drug conjugate**								
**CD30**	Brentuximab vedotin	NCT04609566	II	NSCLC Melanoma	mPFS was 4.1 months (mo) and mOS was 13.9 mo (NSCLC).	Grade ≥ 3 TEAEs in 56%, TESAEs in 42%. Peripheral neuropathy reported in 48% of pts.; 17% of pts discontinued treatment due to TEAEs.	In combination with pembrolizumab Recruiting	[29]
**Bispecific antibodies**								
**CD16A and EGFR**	AFM24	NCT05109442	I/IIa	NSCLC	3/15 PR, 1/15 CR, 7/15 SD.	2/17 Grade 3 toxicity infusion-related reaction.	In combination with atezolizumab EGFR wild type Ongoing study, recruiting	[30]
**PD-L1 and** **4-1BB**	Acasunlimab	NCT05117242	II	NSCLC	ORR 13%, 21%, and 22% with 6 mo PFS 0%, 18%, and 33% for monotherapy, pembrolizumab combination therapy q3 week, or pembrolizumab combination therapy q6 week.	Most common grade ≥ 3 TRAEs were asthenia, liver-related events, and anemia.	Patients progressed on ICI Monotherapy and combination therapy with pembrolizumab Active, not recruiting	[31]
**PD-L1 and CTLA-4**	KN046	NCT04054531	II	NSCLC	ORR is 46.0%, and the median duration of response is 8.1 mo. The mPFS and mOS are 5.8 and 26.6 mo, respectively.	Grade ≥ 3 TRAEs was observed to be 66.7%. Treatment-related deaths occurred in 4 (4.6%) patients, with one case of immune-related pneumonia attributed to KN046.	First-line In combination with chemotherapy	[32]
**PD-L1 and VEGF**	HB0025	NCT04678908	I	NSCLC	ORR 25%, DCR 66.7% with 3/12 PR, 5/12 SD, and 4/12 PD.	11/12 patients experienced TRAE. Grade 3 ≥ TRAEs occurred in 2/12. No DLT.	Monotherapy Included TKI-resistant population with EGFR/ALK mutation	[33]
**PD-L1 and VEGF**	PM8002	NCT05918445	Ib/Iia	NSCLC	16/61 PR, 32/61 SD.	11/61 experienced grade ≥ 3 TRAEs. 5/61 patients discontinued due to TRAEs.	Monotherapy Included TKI-resistant population with EGFR mutation	[34]
**CD137 and PD-L1**	MCLA-145	NCT03922204	I	Advanced solid tumors	DCR 37% with monotherapy and 68% with combination.	6/53 patients receiving monotherapy had DLTs. No DLTs occurred in the patients receiving combination therapy.	Monotherapy or in combination with pembrolizumab	[35]
**Cellular therapy**								
**KRAS**	FH-A11KRASG12V-TCR	NCT06043713	I	mutant KRAS including pancreatic, colorectal, and NSCLC	-	-	Recruiting	[36]
**Tumor-infiltrating lymphocytes (TILs)**	GT201	NCT05729399.	I	Advanced solid tumors	3/7 patients PR, 2/7 patients SD. In the NSCLC subgroup, disease control was observed in 3/3 patients.	Grade ≥ 3 AEs related to lymphodepleting chemotherapy and IL-2 included decreased lymphocyte count, decreased neutrophil count, and decreased white blood cell count, pyrexia and increased heart rate.	3/7 patients PR, 2/7 patients SD. In the NSCLC subgroup, disease control was observed in 3/3 patients Recruiting	[37]
**Microbiome-based therapeutics**								
**Gut** **microbiome**	BMC128	NCT05354102	I	RCC, melanoma, and NSCLC	4/8 patients with SD.	No SAEs.	In combination with nivolumab	[38]
**Gut** **microbiome**	hdFMT	NCT05669846	II	NSCLC	-	-	Patients with R/R NSCLC progressed on prior anti-PD-1-based therapy Not yet recruiting	[39]
**Monoclonal antibodies**								
**anti-ILT3**	MK-0482	NCT04165798	II	NSCLC	ORR (95% CI) 4% (1%−15%; 0 CR, 2 PR). PFS was 2.6 (1.4−4.7) mo.	Grade ≥ 3 in 17 patients (38%).	In combination with pembrolizumab	[40]
**anti-ILT4**	MK-4830	NCT04165798	II	NSCLC	ORR (95% CI) 11% (4−24%; 1 CR, 4 PR). PFS was 2.4 (1.5−2.7) mo.	Grade ≥ 3 in 20 (44%).	In combination with pembrolizumab	[40]
**anti-NKG2A/CD94**	monalizumab	NCT02671435	I/II	Advanced solid tumors	2/20 PR and 6/20 SD.	No DLTs.	In combination with durvalumab	[41]
**CD27 agonist**	boserolimab	NCT04165798	II	NSCLC	ORR (95% CI) was 8% (2−22%; 1 CR, 2 PR). PFS was 2.4 mo.	Grade ≥ 3 in 21 (57%).	In combination with pembrolizumab	[40]
**Complement factor H (CFH)**	GT103	NCT04314089	Ib	NSCLC	Best treatment response was SD in 9/31 patients.	3/31 patients experienced grade ≥ g3 TRAEs	Monotherapy	[42]
**Complement factor H (CFH)**	GT103	NCT05617313	II	NSCLC	-	-	In combination with pembrolizumab Ongoing	
**CTLA-4**	IBI310	NCT05118334	II	NSCLC	2/30 PR, 15/30 SD, 2/20 ORR, 17/30 DCR.	TEAEs leading to treatment interruption of any treatment drug occurred in 7 (7/15, 46.7%) and 9 (9/15, 60%) patients in cohorts A and B, respectively. Three treatment-related deaths.	In combination with sintilimab (anti-PD1)	[43]
**LAG-3**	LBL-007	NCT05102006	Ib/II	Advanced solid tumors	Out of 75 efficacy evaluable patients, ORR and DCR were 13.3% and 48.0%, respectively.	Treatment interruption and permanent discontinuation due to TEAEs occurred in 6 (7.5%) patients each.	In combination with toripalimab (anti-PD-1)	[44]
**LAG-3**	Ieramilimab	NCT02460224	II	Advanced solid tumors	In NSCLC cohort, 3/42 PR, 18/42 with SD, ORR 15%.	In the anti-PD-1/L1 naive and pretreated cohorts, 9.9% and 5.4% of patients, respectively, discontinued study treatment due to AEs regardless of study drug relationship.	In combination with startalizumab (anti-PD-1)	[45]
**Other**								
**CXCR2**	Navarixin	NCT03473925	II	Advanced solid tumors	mPFS was 1.8–2.4 mo without evidence of a dose–response relationship, and the study was closed at a prespecified interim analysis for lack of efficacy.	DLTs occurred in 2/48 patients (4%) receiving navarixin 30 mg and 3/48 (6%) receiving navarixin 100 mg.	In combination with pembrolizumab	[46]
**TLR7/8**	EIK1001	NCT06246110	II	NSCLC	-	-	In combination with pembrolizumab Recruiting	[47]
**α/β IL-2R**	STK-012	NCT05098132	Ia/Ib	Advanced solid tumors	3/38 PR. 17/38 SD.	No DLTs.	Monotherapy	[48]
**Cancer vaccines**								
**CCL21**	IT CCL21-DC	NCT03546361	I	NSCLC	-	-	Active, not recruiting	[49]
HLA-A*02:01-restricted **peptides**	PDC*lung01	NCT03970746	I/II	NSCLC	The BOR included 12 PR (63.2%) and 7 SD (36.8%) with ORR of 63.2% (80% CI 45.9–78.2%) and DCR of 94.7%. The mPFS was 10.9 months.	Only 1 severe TRAE occurred, a grade 4 allergic infusion-related reaction, leading to IMP discontinuation.	In combination with pembrolizumab HLA-A*02 positive	[50]
**Tumor** **associated** **antigen**	CAN-2409	NCT04495153	II	NSCLC	mOS of evaluable population was 22.0 mo (*n* = 44). 64% had systemic clinical response of injected and uninjected lesions.	No DLT.	Refractory or resistant to ICI Injected into tumor followed by oral prodrug (valacyclovir)	[51]
**Tumor** **associated** **antigen**	BNT116	NCT05142189	I	NSCLC	7/20 (35%) PR, 10/20 (50%) SD. ORR was 35% (95% CI: 15.4–59.2%) and the DCR was 85% (95% CI: 62.1–96.8%).	TEAEs ≥ grade 3, (incidence rate ≥10%). No DLTs within the dose confirmation period or deaths under treatment were observed.	In combination with docetaxel	[52]
**Tumor** **associated** **antigen**	BI1361849	NCT03164772	IB	NSCLC	Arm A had 29% ORR and 71% DCR, median months of 10, 7.3 5.7, and not reached for DOR, irPFS, PFS, and OS, respectively. In contrast, arm B yielded 11% ORR and 53% DCR, median months of 6, 2.5, 2.5 and 10 for DOR, irPFS, PFS, and OS, respectively.	Both arms had comparable TRAEs (56–57%) of a generally low grade and manageable within current guidelines. Both arms had a comparable rate of treatment discontinuation (22–24%).	Arm A: BI1361849 + durvalumab and arm B: BI1361849 + durvalumab+ tremelimumab	[53]

AEs, adverse events; BOR, best overall response; CI, confidence interval; CR, complete response; DCR, disease control rate; DLT, dose-limiting toxicity; DOR, duration of response; hdFMT, healthy donor fecal microbiota transplant; ICI, immune checkpoint inhibitor; irPFS, immune-related progression-free survival; mOS, median overall survival; mPFS, median progression-free survival; ORR, overall response rate; OS, overall survival; PD, progressive disease; PFS, progression-free survival; pts, patients; PR, partial response; q3, every three; q6, every six; SAEs, serious adverse events; SD, stable disease; TESAEs, treatment-related serious adverse events; TKI, tyrosine kinase inhibitor; TRAEs, treatment-related adverse events.

## Data Availability

No new data were created or analyzed in this study. Data sharing is not applicable to this article.
